# A Magnetic Sensor with Amorphous Wire

**DOI:** 10.3390/s140610644

**Published:** 2014-06-17

**Authors:** Dongfeng He, Mitsuharu Shiwa

**Affiliations:** National Institute for Materials Science, 1-2-1 Sengen, Tsukuba, Ibaraki 305-0047, Japan; E-Mail: SHIWA.Mitsuharu@nims.go.jp

**Keywords:** magnetic sensor, amorphous wire, FeCoSiB

## Abstract

Using a FeCoSiB amorphous wire and a coil wrapped around it, we have developed a sensitive magnetic sensor. When a 5 mm long amorphous wire with the diameter of 0.1 mm was used, the magnetic field noise spectrum of the sensor was about 30 pT/√Hz above 30 Hz. To show the sensitivity and the spatial resolution, the magnetic field of a thousand Japanese yen was scanned with the magnetic sensor.

## 1. Introduction

Many kinds of high sensitive magnetic field sensors have been developed. Among them, the inductive coil sensor [1] is one of the most commonly used magnetic types. A highly sensitive inductive coil sensor with a noise level around 50 fT/√Hz at 10 kHz was fabricated using amorphous ribbon (Metglas 2714AF) with length of 150 mm, cross section of 5 × 5 mm^2^, and a coil of 10,000 wound turns [2]. However, inductive coil sensors cannot measure the DC magnetic field and it is difficult to obtain low noise levels at low frequency with small inductive coil sensors.

A fluxgate magnetometer can measure the DC magnetic field [3]. It consists of three coils wound around a ferromagnetic core: an AC excitation winding, a detection winding that indicates the zero field condition and a DC bias coil that creates and maintains the zero field. The use of modern materials for magnetic cores has improved the sensitivity of fluxgate magnetometers to about several pT/√Hz [4], but the operation frequencies of flux gate magnetometers are normally low, which limits their measuring bandwidth.

Giant magnetoresistive (GMR) sensors [5], anisotropic magneto resistive (AMR) sensors [6], and magneto-impedance (MI) sensors [7,8] can measure DC magnetic fields and have big measuring bandwidth. They have been developed and used in various areas like nondestructive evaluation, communication, geological exploration, medical diagnostics, security control, *etc*. [9–12]. Especially for the pulse-driven magnetoimpedance (PMI) sensor, pico-Tesla (pT) level magnetic field resolution has been obtained [13] and the sensors were used as biosensors to measure the biomagnetic fields in musculatures with spontaneous electric activity [14], biomagnetic activity in the heart [15] and anticancer drugs (curcumin) tagged to superparamagnetic (Fe_3_O_4_) nanoparticles [16].

The sensors of GMR sensors, AMR sensors and MI sensors normally have electrical connections with the sensing parts, which are not convenient to fabricate in some applications, such as the construction of magnetic microscopes, where a small distance of several micrometers between the sensor and the sample is needed. In this paper, we will describe a small simple sensitive magnetic field sensor using (Fe_0.06_Co_0.94_)_72.5_Si_2.5_B_15_ (FeCoSiB) amorphous wire with a coil wrapped around it.

## 2. Analysis of the Magnetic Sensor

**Figure 1.**
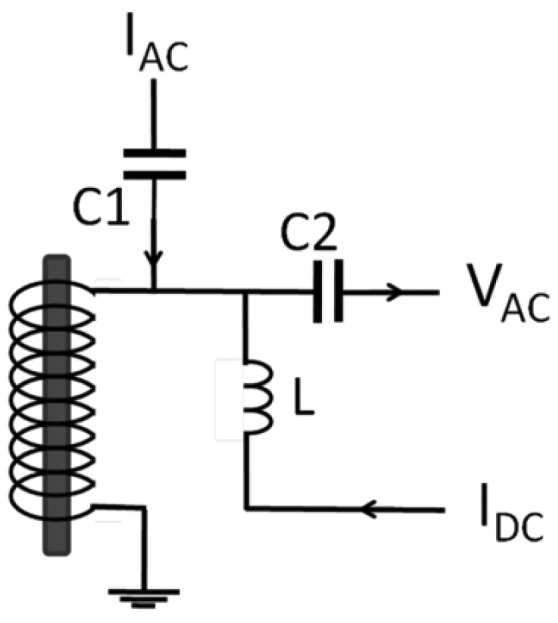
Configuration of the magnetic sensor.

Figure 1 shows the configuration of the magnetic sensor, which is composed by a coil and a FeCoSiB amorphous wire. An AC current and a DC current flow in the coil to produce the AC modulation magnetic field and DC bias magnetic field. The capacitors C1, C2 and the inductor L are used to isolate the DC current or AC current. When a proper DC bias field is applied to the sensor, due to the nonlinearity of the B-H curve of the amorphous wire, the amplitude of the AC voltage V_AC_ changes with the external field. This is the principle of the magnetic sensor.

The inductance of the coil in Figure 1 can be estimated by following formula [17]:

(1) $$L=\frac{k\mu_{0}\mu_{re}N^{2}A}{l}$$

where, *L* is the inductance of the coil; *l* is the length of the coil; *N* is the turns of the coil; *A* is the cross area of the amorphous wire; *μ*_0_ is the vacuum magnetic permeability; *μ_re_* is the effective relative magnetic permeability, which is related with the permeability of the amorphous wire, the diameter of the amorphous wire and the diameter of the coil; *k* is a constant factor determined by the geometry of the coil. For the frequency *ω*, the impedance *Z* of the coil can be expressed as:

(2) $$Z=j\omega L=\frac{j\omega k\mu_{0}\mu_{re}N^{2}A}{l}$$

If a single frequency current source *I_AC_* = *Ie^jωt^* flows in the coil; the voltage *V_AC_* across the coil can be expressed:

(3) $$V_{AC}=I_{AC}Z\approx \frac{j\omega k\mu_{0}\mu_{re}N^{2}AI}{l}e^{j\omega t}=Vje^{j\omega t}$$

(4) $$V=\frac{\omega k\mu_{0}\mu_{re}N^{2}AI}{l}$$

where, *I* is the amplitude of *I_AC_* and *V* is the amplitude of *V_AC_*. Due to the nonlinearity of the M-H curve of FeCoSiB amorphous wire, the effective relative permeability of the amorphous wire *μ_re_* = *əB/əH* changes with the external magnetic field, then the amplitude of *V_AC_* also changes with the external field *H*.

## 3. Experiments and Results

**Figure 2.**
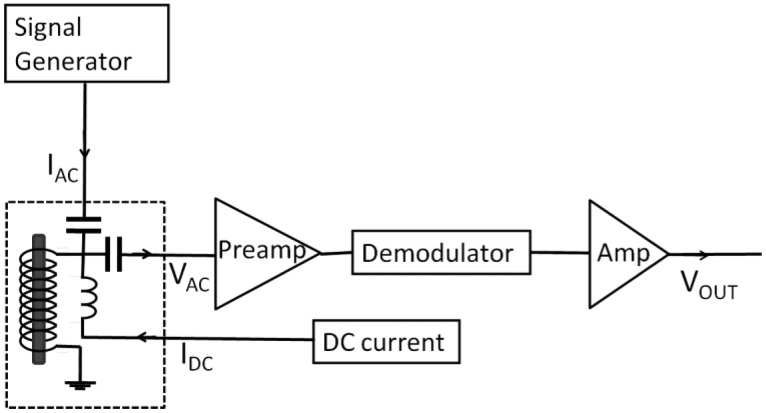
Block diagram of the driving circuit of the magnetic sensor.

Figure 2 shows the block diagram of the driving circuit of the magnetic sensor. A 5 mm-long FeCoSiB amorphous wire with the diameter of 100 μm is used, which is made by UNITIKA Ltd. (Nagoya, Japan) using water quenched spinning method.

For this FeCoSiB amorphous wire with the ratio of Fe (0.06) and Co (0.94), the magnetostriction value λ_S_ is close to zero [18], and the B-H curve is steep with small hysteresis [19]. The saturation magnetization of the wire is about 0.81 T with relative permeability at zero magnetic field of about 2000. The saturation magnetic field is about 300 A/m. The wrapped coil is 30 turn single layer coil. The diameter D of the coil is about 0.6 mm. The signal generator is used to supply a sine wave current. In our experiments, 1 MHz sine wave current is used and the amplitude is about 20 mA. A DC current is used to produce the bias DC magnetic field, which is necessary to achieve the best operation of the magnetic sensor. The AC voltage across the coil is amplified by a preamplifier. A demodulator is used to get the amplitude of the AC voltage. After the demodulator, an amplifier is used and the output DC voltage is adjusted to zero when there is no external magnetic field. The signal of V_OUT_ corresponds to the external magnetic field.

**Figure 3.**
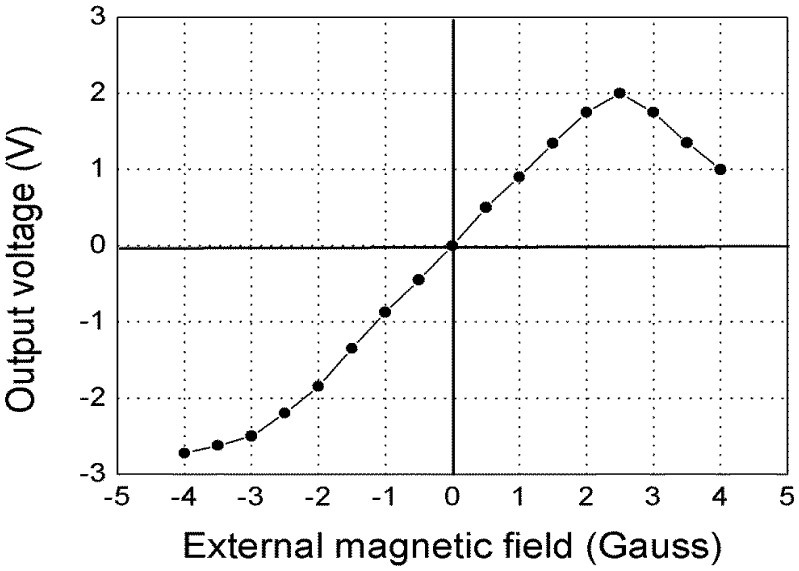
Output voltage changes with the external magnetic field when the static bias magnetic field is about 2.5 Gauss.

Figure 3 shows the output voltage changes with the external magnetic field when the bias DC magnetic field is about 2.5 Gauss. For external magnetic field between −2 Gauss and +2 Gauss, the magnetic field response is nearly linear. The output voltage was about 0.9 V/Gauss.

**Figure 4.**
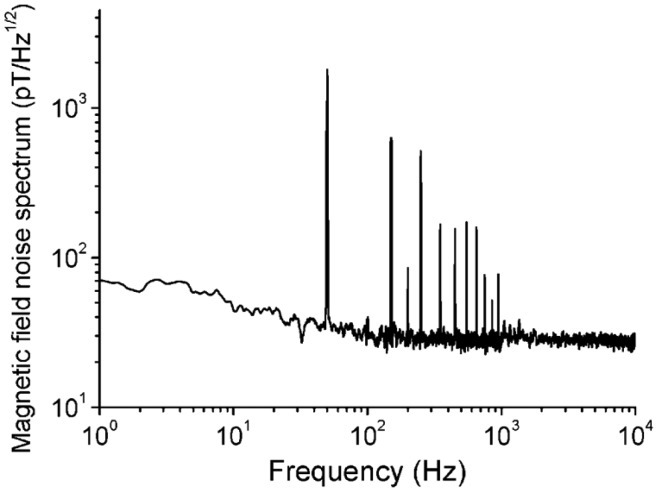
Magnetic field noise spectrum of the magnetic sensor measured in a 1 mm one layer permalloy shielding box.

To measure the magnetic field noise spectrum, we first measure the noise spectrum of the output voltage of the GMI sensor using a spectrum analyzer, then divide it by the value of 0.9 V/Gauss obtained from Figure 3. Figure 4 shows the magnetic field noise spectrum of the magnetic sensor measured in a 1 mm one layer permalloy shielding box. The peaks are the 50 Hz inference and its harmonics. The white magnetic field noise spectrum is about 30 pT/√Hz.

## 4. Application and Discussion

**Figure 5.**
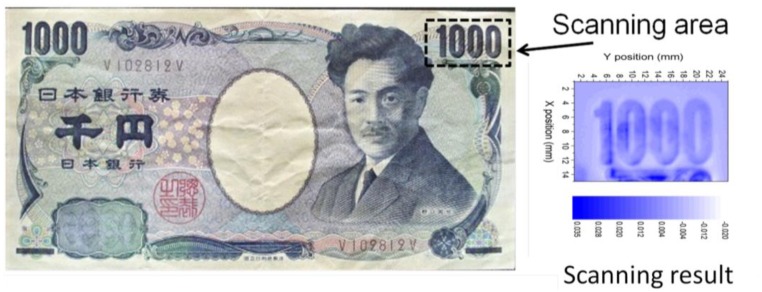
Scanning result of Japanese thousand bill using the magnetic sensor.

Due to the small diameter of the amorphous wire, this magnetic sensor can be used to construct a magnetic microscope. To prove the sensitivity and the spatial resolution of the sensor, we measured the magnetic field produced by a Japanese thousand yen bill, which is printed with magnetic ink. The bill is put on an X-Y stage for the scanning with scanning steps of 0.1 mm. The lift off between the bill and the sensor is about 0.1 mm. The measurement is done in an unshielded environment. Figure 5 shows the scanning result. The number 1000 is clearly observed.

Because the magnetic properties of FeCoSiB amorphous wire change with the temperature, the variance of environmental temperature will cause low frequency drift of the sensor. In our scanning measurements of the thousand yen bill and eddy current testing using the sensor, the influence is small because the measuring time is not long. In the future, we will develop a bridge type magnetic sensor to reduce the influence of the variance of environmental temperature.

## 5. Conclusions

A simple small high sensitive magnetic sensor with FeCoSiB amorphous wire was developed. This sensor can be used for magnetic microscope and eddy current nondestructive evaluation.
